# Supplementary material for: Recycling Polyethylene/Polyamide Multilayer Films with Poly(isoprene-g-Maleic Anhydride) Compatibilizer
Source: Polymers (Basel). 2024 Apr 12;16(8):1079. doi: 10.3390/polym16081079 (PMC11053548; doi:10.3390/polym16081079)
Supplement: Supplementary file 1 [file polymers-16-01079-s001.zip › polymers-2868286-supplementary.pdf]

Supporting Information

Recycling Polyethylene/Polyamide Multilayer Films with Poly(isoprene-*g*-Maleic Anhydride) Compatibilizer

Andreia Romeiro [1], Cidália Teixeira [2], Henrique Costa [3], Jorge F. J. Coelho [1] and Arménio C. Serra [1,*]

[1] University of Coimbra, Centre for Mechanical Engineering Materials and Processes, ARISE, Department of Chemical Engineering, Rua Silvio Lima- Polo II, 3030-790 Coimbra, Portugal; andreiar@eq.uc.pt (A.R.); jcoelho@eq.uc.pt (J.F.J.C.)

[2] Componit, lda, Estrada Nacional 3 km 28.6, Vila Chã de Ourique, 2071-621 Santarém, Portugal; cidalia.paula@isolago.com

[3] Inventive Matl, Casal dos Eucaliptos, Casais Lagartos, 2070-389 Pontével, Portugal; incentivos@inventivemat.pt

* Correspondence: aserra@eq.uc.pt

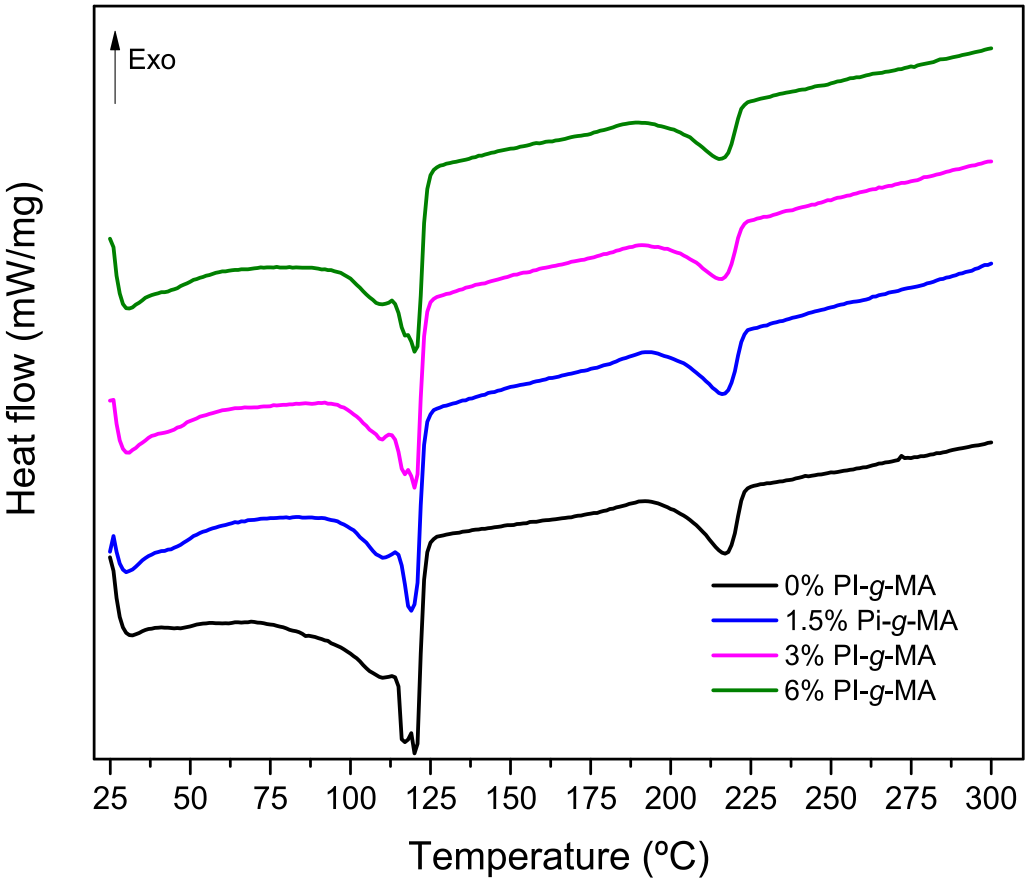

Figure S1 DSC thermogram of 1st heat flux of films with different percentages of PI-*g*-MA.

Table S1 DSC data of the 1st heat flux of the films with different percentages of PI-*g*-MA.

**Sample**	**Polyethylene**				**Polyamide**			
	**T_m (°C)**	**ΔH_m (J/g)**	**Tc (°C)**	**ΔHc (J/g)**	**T_m (°C)**	**ΔH_m (J/g)**	**Tc (°C)**	**ΔHc (J/g)**
**0% PI-*g*-MA**	120	108.5	108	55.2	216	81.5	175	48.0
**1.5%PI-*g*-MA**	118	95.5	107	71.1	216	99.9	172	41.0
**3%PI-*g*-MA**	120	96.1	107	61.7	215	74.2	168	35.1
**6%PI-*g*-MA**	120	100.2	107	64.7	215	78.9	168	32.2
